# The Contribution of Non-Neuronal Cells in Neurodegeneration: From Molecular Pathogenesis to Therapeutic Challenges

**DOI:** 10.3390/cells11020193

**Published:** 2022-01-06

**Authors:** Nadia D’Ambrosi, Mauro Cozzolino, Savina Apolloni

**Affiliations:** 1 Department of Biology, University of Rome Tor Vergata, 00133 Rome, Italy; 2 Institute of Translational Pharmacology, CNR, 00133 Rome, Italy

Neuron loss occurring in neurodegenerative diseases represents just the final step in a series of events involving several cell types, other than neurons, that actively contribute to the overall pathogenic mechanisms by establishing harmful non-cell autonomous effects [1]. Astrocytic and microglial cell activation, oligodendrogliopathy, blood–brain barrier permeabilization, peripheral blood cell infiltration, muscle and adipose tissue alterations often occur before detectable neuron loss and overt symptoms, suggesting their early implication in circumstances that could be considered causal to the disease. 

Consequently, it is of extreme importance to understand if and how the intervention on non-neuronal targets could halt pathological features, representing a potential mainstream or complementary therapy to contrast such complex pathologies.

In this Special Issue of *Cells*, entitled “The Contribution of Non-Neuronal Cells in Neurodegeneration: From Molecular Pathogenesis to Therapeutic Challenges”, 13 original and review articles were published. Here, we will present a summary of the most relevant topics and results arising from this Special Issue, which certainly will increase our knowledge of the role of non-neuronal cells in neurodegeneration, both at the cellular and molecular levels, and will help defining potential therapeutic targets to fight neurological diseases.

The published papers discuss the contribution of astrocytes, microglia, oligodendrocytes and muscle cells in the pathogenesis of neurodegenerative diseases such as amyotrophic lateral sclerosis (ALS), multiple sclerosis (MS), Alzheimer’s disease (AD) and Huntington’s disease (HD).

In the last few years, our knowledge of the importance of glial cells for maintaining central nervous system homeostasis in health conditions has increased exponentially, along with our awareness of their fundamental and multifaceted role in pathological conditions. Among glial cells, astrocytes emerged as promising therapeutic targets in various neurodegenerative disorders. Valori et al. [2], discussed how the increased comprehension of the extraordinary properties of astrocytes offers unprecedented therapeutic opportunities to modulate the activities of distinct astrocyte subpopulations during diseases, to achieve control over neurodegeneration. The review also explores the potential beneficial effect of astrocyte replacement and the challenges and recent advancements in developing astrocyte-specific delivery systems. The study of astrocytes in the context of a specific neurodegenerative disease, AD, is presented by Premam and colleagues [3]. In their paper, the authors discussed how the advent of multi-omic approaches enabled a rapid progress in the characterization of distinct pathological astrocyte states in specific stages of AD, unveiling the opportunity to exploit novel biomarkers and targets for therapeutic intervention.

Aside from astrocytes, microglia cells also participate in the homeostatic modulation of the central nervous system and neuronal function, for instance by regulating the elimination (or “pruning”) of weaker synapses in both physiologic and pathologic processes. Geloso and D’Ambrosi discussed the evidence of microglial-dependent synapse elimination in primitive grey matter damage as an emerging and important contributor to MS patients’ long-term disability, associated with early and progressive cognitive decline [4]. Ulland and colleagues instead proposed that microglial activation, via an inflammasome-dependent mechanism, links AD pathology to sleep breathing disorder (SBD), possibly leading to the reciprocal and synergistic exacerbation of both diseases [5]. The role of microglia was also examined by Fernandes et al., who in their research article, analyzed the temporal profile of inflammatory mediators and microglia deactivation/activation in the brain cortex and hippocampus of a mouse model of AD, highlighting divergent microglia phenotypes and a loss of homeostatic properties that precede AD onset [6]. These data provide the possibility of therapeutic intervention that may restore the imbalance of microglia activation, preventing the progression of the disease. A further proof of the interplay between inflammation and neurodegeneration was demonstrated by Paldino et al., in a model of HD [7]. The authors, in their research article, showed that the administration of Olaparib, an inhibitor of PARP, increased survival, ameliorated neurological deficits, and improved clinical outcomes in neurobehavioral tests, mainly by modulating the inflammasome activation in microglia cells.

In ALS, the neuroinflammatory processes mediated by activated astrocytes and microglia play a relevant role but the link between the dysregulation of RNA metabolism, a central event in the degeneration of motor neurons, and neuroinflammation is poorly defined. To this end, Rossi and Cozzolino discussed the available evidence, showing that RNA-binding proteins (RBPs) and associated RNA processing are affected in ALS astrocytes and microglia, and the possible mechanisms involved in these events [8]. In the context of aberrant pathological mechanisms sustained by glial cells, the deregulation of mRNA transport and of localized translation, a crucial mechanism that regulates proper protein homeostasis in subcellular compartments, which were extensively studied in neurons, is now emerging. In this regard, Blanco-Urrejola et al. thoroughly discussed the possibility that the local translation in glia could contribute to neuronal dysfunction in many neurological and neurodegenerative diseases [9].

Regarding other important players in the neurodegenerative pathogenesis, i.e., oligodendrocytes, Raffaele et al., focused their review on the role of dysfunctional oligodendrocytes in ALS pathogenesis, examining the possible mechanisms involved, ranging from cell degeneration to defective oligodendrocyte precursor cells maturation, and impairment in the energy supply to motor neurons [10]. On this basis, the authors discuss new therapeutic perspectives for ALS treatment based on novel drugs able to target neuroinflammation, which are capable of implementing the remyelinating potential of oligodendrocytes as well as enhancing their energy metabolism. Crabe et al. point to the degeneration of interneurons, in addition to astrocytes and oligodendrocytes, as a key event in disrupting the functional environment of motor neurons, thus sustaining the idea that targeting this pathogenic cellular network represents a novel strategic field of therapeutic investigation in ALS [11]. 

Finally, Scaricamazza et al. reviewed the most recent clinical and preclinical studies in ALS, focusing on another tissue, whose function in the pathogenesis of ALS is still a matter of debate, i.e., the skeletal muscle, considered as the main determinant of the whole-body energy expenditure [12]. The authors underline that, since the functions of muscles and motor neurons are tightly intertwined, the therapeutic interventions that improve skeletal muscle metabolism ultimately may protect motor neurons. A central role of the muscle as a possible therapeutic target in ALS, was also proposed in the research article by Ceccanti et al. [13]. The authors indeed demonstrate that higher levels of serum creatinine and myoglobin, two muscular metabolic and oxygen reservoirs, respectively, are linked to a slow progression of ALS, and thus can represent a useful tool to predict and monitor disease progression. 

Lastly, D’Ambrosi et al., provided a comprehensive review of the role of S100A4, a calcium-binding protein exerting a broad range of functions, focusing on the pathophysiology of the nervous system [14]. They described that, by affecting the functions of astrocytes, microglia, infiltrating cells and neurons, S100A4 regulates inflammation and immune reactions and modulates neuronal plasticity and survival, thus representing a target of potential interest for clinical applications.

In summary, this Special Issue presents 13 articles that help us to improve our knowledge of the role of non-neuronal, cell-related mechanisms in the onset and progression of neurodegeneration, and provide further support to the concept that targeting these cells represents a possible therapeutic strategy to counteract neurological conditions.
